# Crb2-dependent progenitor adhesion safeguards ventricular lining integrity and prevents hydrocephalus

**DOI:** 10.21203/rs.3.rs-10244145/v1

**Published:** 2026-08-13

**Authors:** Aikaterini Dimou, Raehee Park, Olivia M. Pericak, Minjung Lee, Michael J. Holtzman, Seo-Hee Cho, Seonhee Kim

**Affiliations:** Temple University; Temple University; Temple University; Temple University; University of Washington; Thomas Jefferson University; Temple University

**Keywords:** Congenital hydrocephalus, CRB2, neural progenitor, adherens junction, ependymal cell

## Abstract

Congenital hydrocephalus is a heterogeneous neurodevelopmental disorder in which ventricular enlargement can arise from impaired cerebrospinal fluid (CSF) flow, aqueductal obstruction, or altered ventricular wall integrity. Although human CRB2 mutations are associated with hydrocephalus, aqueductal abnormalities, and periventricular heterotopia, the relevant cell types and pathogenic sequence remain poorly defined. Here, using lineage-specific Crb1/2 conditional mouse models, we show that Crb2-dependent polarity and adhesion in neural progenitors are required to preserve ventricular system integrity. Pan-neural Crb1/2 deletion recapitulated major features of CRB2-associated disease, whereas deletion in ependymal lineage-specified progenitors did not disrupt ependymal maintenance or cause ventriculomegaly. Cortical progenitor-specific deletion caused progressive ventriculomegaly and impaired CSF transit despite a patent, ciliated aqueduct and largely intact neurogenesis. Mechanistically, Crb2 loss disrupted apical polarity, adherens junction continuity, actomyosin organization, and ventricular barrier integrity, leading to ventricular lining detachment and increased permeability to ventricular contents. Furthermore, intracerebroventricular administration of bumetanide attenuated ventricular enlargement without restoring ependymal organization, indicating that CSF load contributes to ventricular expansion when the ventricular wall is mechanically weakened. Importantly, detached rostral progenitor/ependymal precursor can be preserved at the blocked caudal aqueduct, providing evidence of unaffected ependymal cell generation without Crb1/2. These findings define CRB2-associated hydrocephalus as a progenitor-stage adhesion and ventricular wall integrity disorder.

## Introduction

Hydrocephalus (HC) is characterized by pathological enlargement of the cerebral ventricles due to the accumulation of cerebrospinal fluid (CSF), which can increase intracranial pressure and damage brain parenchyma in both humans and animal models(1). Depending on the presence of physical blockage in CSF flow, congenital hydrocephalus (CH) can be classified as non-communicating (obstructive) or communicating(2, 3). While the cerebral aqueduct, a narrow passage between the third and fourth ventricles, represents a major site of obstruction, imbalance of CSF production and absorption, ineffective CSF flow, and defective compliance of the brain wall are implicated in communicating HC(4, 5). Disruption of the ventricular system, which includes the ventricular lining cells and choroid plexus (ChP), the CSF-producing organ, is associated with HC(6). Pleiotropic effects of a multidimensional pathogenic process can obscure the primary pathogenic mechanisms from their secondary consequences(7–9). Furthermore, the modeling of genetically intractable systems is limited in their ability to recapitulate human phenotypes, hindering our understanding of HC pathogenesis(10, 11). As invasive surgical intervention with a risk of lifelong disability is currently the only treatment option(12, 13), it is imperative to learn more about pathogenic mechanisms with disease-relevant models.

CH affects approximately 1 per 1,000 live births, yet pathogenic mechanisms remain poorly understood despite more than 100 loci having been implicated(7, 10, 14, 15). Human genetic studies increasingly implicate neurodevelopmental pathways in CH, including genes involved in cell signaling, proliferation, polarity, adhesion, and ventricular surface integrity. However, most ventricular system cells are generated from neural progenitors, which complicates the identification of cell-type-specific effects and the sequence of pathogenic mechanisms. Among CH-associated genes, mutations in CRB2 and genes encoding its interacting proteins (e.g., MPDZ) are of particular interest due to their prominent role in cell polarity and adhesion. CRB2 mutations have been linked to CH in humans and, in a subset of patients, to aqueductal stenosis, affected individuals also present with ventriculomegaly and cortical malformations, such as periventricular heterotopias (PH). However, it is unclear whether this pathology can be recapitulated in animal models and which cell types are the primary drivers of pathogenic cascades. To overcome this hurdle, we used multiple cell-type-specific Cre drivers to delete Crb2 and study cell-type-specific contributions to CH.

*CRB2* encodes a core component of the Crumbs apical polarity complex, forming a tripartite complex with PALS1 (protein associated with Lin7) and MPDZ/INDL1, and localizes to the apical junction(16–19). In mammals, CRB proteins (CRB1, CRB2, CRB3) participate in the Crumbs polarity complex and support apico-basal polarity establishment, apical domain specification, ciliogenesis, and cell adhesion(19). Although the MPDZ mutation causes human CH, and the mouse model demonstrates hydrocephalus in both global knockout and Nestin-Cre-mediated deletion, the cellular mechanism that requires other members of the Crumbs complex remains incompletely understood(20–23). Despite changes in ChP epithelial permeability altering CSF secretomes, Crb3 expression remained intact in the ChP epithelium in the MPDZ global knockout(20). This suggests that although similar human patient outcomes and physical and functional interactions between MPDZ and Crb proteins are known, distinct or unknown pathogenic mechanisms may exist. Therefore, although the genes implicated in HC include those encoding cell polarity complex proteins, junction-associated proteins, and adhesion molecules, it is not clear whether CRB2-dependent HC shares previously recognized mechanisms.

Ventricular integrity in the developing neuroepithelium is supported by proliferating radial glial cells, the principal neural progenitors in the ventricular zone that generate neurons and glia(24–26). During neurogenesis, radial glia cells also generate ependymal cell (EC) precursors, which mature over a prolonged period and give rise to multiciliated EC that line the brain’s ventricles(24, 27, 28). Highly polarized radial glia cells form the ventricular lining by joining apical endfeet via the apical junctional complex (AJC), which comprises adherens junction (AJ) and apical polarity complex proteins such as Crumbs complex. Although the importance of AJC in structural barriers and as a hub for mechanical tension-dependent cellular processes, including mitogenic signaling, dynamic adhesion, and cellular morphogenesis, the impact of AJC disruption on hydrocephalus pathogenesis remains unclear. CRB proteins are highly implicated in adhesive cell-cell interactions at the AJC through extracellular domain-mediated adhesive interactions and AJ formation and maintenance by regulating actomyosin through Rho-ROCK signaling(24, 29, 30). The Crb complex plays a context-dependent regulatory role in Rho-Rock proteins, and the role of Crb2 in AJC integrity is well established. However, the timeline of Crb-dependent AJC defects on ventriculomegaly pathogenesis is not yet known.

In our genetic study, we use cell-type-specific Crb1/Crb2 conditional mouse models to recapitulate key human phenotypes associated with CRB2 mutations and define how Crb1/Crb2 loss disrupts ventricular system development. By targeting broad neural progenitors, cortical progenitors, midbrain aqueduct lineages, and lineage-specified multiciliated cells, including ECs, we distinguish the relative contributions of progenitor polarity, aqueduct architecture, and ependymal maturation to hydrocephalus pathogenesis. These models show that Crb1/Crb2 function primarily in neural progenitors to preserve AJC integrity, ventricular wall cohesion, and barrier function, while cortical progenitor proliferation and ECs maturation are largely preserved. Importantly, midbrain-lineage deletion using Wnt1-Cre2 provides a developmental context to examine CRB2-associated aqueductal pathology and shows that multiciliated ECs and subcommissural organ (SCO) cells can still be generated despite aqueduct disruption, supporting a role for Crb1/Crb2 in ventricular wall stability and cell retention rather than fate specification. Together, our findings identify CRB2-associated hydrocephalus as a progenitor-stage adhesion and ventricular wall integrity disorder rather than a simple consequence of failed ependymal specification or aqueduct obstruction.

## Methods

### Animals

All animal procedures were approved by the Institutional Animal Care and Use Committee of the Lewis Katz School of Medicine at Temple University (protocol #5059) and conducted in accordance with institutional and NIH guidelines. Food and water were provided ad libitum, and mice were maintained on a 12-h light/dark cycle at 70–74°F and 30–70% relative humidity. For these studies, we used CRB-dko^Emx1^, CRB-dko^Nes^, CRB-dko^Foxj1^ and CRB-dko^Wnt1-Cre2^ mice. For each experiment, 3–8 animals per genotype were analyzed. Because sex differences do not affect cortical development at embryonic stages in these models, both sexes were used without stratification. Crb1/Crb2 floxed mice were generated as previously described. Emx1-Cre mice were obtained from The Jackson Laboratory (stock #005628), which drives recombination in the Emx1-expressing cortical lineage. Foxj1-Cre mice(38), which target ciliated epithelial and ECs, were generated as previously described. Wnt1-Cre2 mice were obtained from The Jackson Laboratory (Strain #022137). All mice were genotyped by PCR using standard protocols.

### Histology and immunohistochemistry

Mouse brains were fixed in 4% paraformaldehyde (PFA) in PBS at 4°C for overnight, embedded in paraffin and sectioned at 7μm in the coronal planes. For histological analyses, sections were rehydrated and stained with hematoxylin and eosin. For immunohistochemistry, sections were deparaffinized and rehydrated through graded xylene and ethanol, then rinsed in distilled water. Antigen retrieval was performed by boiling sections in sodium citrate buffer (10mM, pH6.0) containing Tween-20 for 30 min, followed by phosphate-buffered saline (PBS) washes. Primary antibodies were applied in blocking solution at 4°C overnight. The next day, sections were washed in PBS and incubated for 3h at room temperature with fluorophore-conjugated secondary antibodies, Alexa Fluor 488-conjugated goat anti-mouse and anti-rabbit, 647 conjugated goat anti-mouse or anti-rabbit (Invitrogen), Cy3-conjugated goat anti-mouse or anti-rabbit (Jackson Immuno Research) at 1:500 in 5% normal goat serum together with Hoechst 33258 (1:500). After four PBS washes, sections were mounted in Fluoromount-G and imaged on a Leica SP8 confocal microscope. Image acquisition and basic processing were performed using LAS AF (Leica) and Photoshop (Adobe).

For vibratome-cut gelatin sections, brains were fixed in 4% PFA, embedded in gelatin, and sectioned on a vibratome. Free-floating sections were washed in PBS, permeabilized with 0.1% Triton X-100, and incubated with Phalloidin staining solution and Hoechst 33258 (1:500) at room temperature (30 min and 10 min, respectively). Sections were washed in PBS and mounted in Fluoromount-G prior to imaging.

### Primary antibodies

The following primary antibodies were used: CTIP2 (1:200; Abcam, ab18465), CUX1 (1:200; Proteintech, 11733-1-AP), N-cadherin (1:500; BD, 610920), NMIIB (1:500; BioLegend, PRB-445P), PALS1 (1:200; Proteintech, 17710-1-AP), pan-CRB (1:200; [Cho et al., 2012]), PAX6 (1:200; BioLegend, 901301), pMLC (1:200; Abcam, ab2480), TBR2 (1:200; Abcam, ab23345), BrdU (1:500; Abcam, ab6326), NeuN (1:200, Millipore, MAB377), β-Catenin (1:500; BD, 610153), S100β (1:200, Abcam, ab52642), Foxj1 (1:500; Invitrogen, 14-996582), Sox9 (1:200, Millipore, AB5535), Adenylyl Cyclase III (1:200 Santa Cruz, sc-588), Arl13b (UC Davis/NIH 75–287), BrdU (1:500; Abcam, ab6326), α-Catenin (1:200, BD, 610193), AFRU (1:1000, polyclonal anti-rabbit, gift from Dr. Guerra, Universidad Austral de Chile).

### FlashTag (CFSE) labeling

To label ventricular progenitors, 2 μl of 1.12mM CellTrace CFSE (CellTrace^™^ CFSE, Invitrogen, Cat No: C34554 A)/1% Fast Green dye solution was injected into the lateral ventricle at E17.5 using a pulled glass micropipette. Embryos were collected at E18.5, and brains were fixed, processed, and imaged by confocal microscopy to assess CFSE distribution.

### BrdU labeling

To assess proliferative populations, time-pregnant females received a single intraperitoneal injection of BrdU (50 mg/kg) at E14.5 or E16.5. Paraffin sections through the rostral aqueduct were prepared and immunostained for BrdU in combination with lineage markers (e.g., Tbr2, Sox9) to identify proliferating intermediate progenitors and glial precursors.

### Dye injection to assess CSF flow

To assess cerebrospinal fluid flow, 4 μl of 1% fast green were injected into the lateral ventricle at P6, P7 and P8 using a pulled glass micropipette. Mice were euthanized 10 min after injection, and brains were examined by microscopy to evaluate dye diffusion from the lateral ventricles to the fourth ventricle and assess evidence of aqueductal blockage.

### BDA injections

For tracer analysis of CSF pathways, 4 μl of 1% fast green dye mixed with a solution of biotinylated dextran amine, BDA (lysine fixable, MW 10,000; Molecular Probes, Eugene, OR), (10% in phosphate buffered saline (PBS), pH 7.4), was injected into the lateral ventricle at P8 by ICV injection using a pulled glass micropipette. Mice were euthanized 20 min after injection, and brains were post-fixed in 4% PFA overnight followed by PBS washes. Gelatin-embedded brains were sectioned on a vibratome. Sections were permeabilized in 0.1% Triton X-100, incubated with streptavidin-conjugated fluorophore (1:200, Streptavidin, Alexa Fluor^™^ 594 Conjugate, Invitrogen) and Hoechst 33258 (1:500) at room temperature (30 min and 10 min, respectively), washed in PBS, mounted in Fluoromount-G, and imaged by fluorescence microscopy to visualize tracer distribution.

### Bumetanide/FlashTag injections

To pharmacologically target NKCC1-mediated ion transport, a bumetanide (Sigma-Aldrich, B3023)/FlashTag working solution was prepared by combining bumetanide stock (50 mg/ml in 100% ethanol; 250 mg in 5 ml) with the FlashTag mix (CFSE stock: 50 μg CellTrace CFSE in 8 μl DMSO, diluted in PBS with a trace of Fast Green) to a final bumetanide concentration of 2 mg/ml. A volume of 2 μl of this solution was injected into the lateral ventricle of P0 pups. Brains were collected at P8 for histological and imaging analyses. Experimental groups included: Bumetanide/FlashTag mix Controls, Bumetanide/FlashTag mix CRB-dko^Emx1^, Ethanol/FlashTag mix (vehicle) Controls, and Ethanol/FlashTag mix (vehicle) CRB-dko^Emx1^.

### Quantification and statistical analysis

Cell quantification, including manual cell counts, was performed on confocal images using Photoshop (Adobe). All data are presented as mean ± SD. unless otherwise indicated. Group comparisons for histological and cell-count data were performed in GraphPad Prism using two-tailed unpaired Student’s t-tests for two-group comparisons or one-way ANOVA and two-way ANOVA followed by appropriate post hoc tests for experiments with more than two groups, as specified in the figure legends. Normality and homogeneity of variance were assessed where applicable, and non-parametric tests were applied when these assumptions were not met. The number of animals or sections (N) for each experiment is indicated in the figure legends. A significance threshold of P < 0.05 was used for all tests. For RNA-seq data, differential expression was assessed using DESeq2, which models count data with a negative binomial distribution and applies Wald tests with Benjamini-Hochberg correction to control the false discovery rate (FDR).

### Bulk RNA sequencing

Bulk RNA-seq libraries were prepared according to Illumina protocols. Sequencing was performed on an Illumina platform, and reads were aligned to the Mus musculus reference genome (UCSC mm10) using the Cufflinks Assembly & DE BaseSpace workflow, which includes Isis analysis software, STAR aligner, BEDTools, Cufflinks, and BLAST. Raw gene-level counts were extracted from Illumina output and imported into RStudio for downstream analysis. Count matrices from individual samples were merged and analyzed using DESeq2 (Bioconductor) for differential expression, following standard RNA-seq workflows.

## Results

### Crb2 deletion in neural progenitors causes early-onset hydrocephalus with periventricular heterotopias.

To study the role of Crb2 in ventricular system development and the cellular and molecular mechanisms underlying hydrocephalus pathogenesis, genetic deletion is conducted with multiple Cre lines and Crb1 and 2 floxed alleles. To eliminate potential variability due to the widely distributed Crb1 mutation, such as *rd8*, in many mouse lines, we used a double-floxed allele to delete both Crb1 and Crb2 in a cell-type-specific manner. Of note, in all Cre lines analyzed, hydrocephalus and associated ventricular defects segregated strictly with Cre-positive mice homozygous for the floxed Crb2 allele. By contrast, animals that were Cre-negative or Cre-positive but retained at least one functional Crb2 allele were phenotypically normal, regardless of their Crb1 genotype. First, we generated CRB-dko^Nes^ mice, in which Crb2 and Crb1 are deleted in most neural progenitors using Crb1/2 floxed mice and Nestin-Cre mice (23, 31). CRB-dko^Nes^ mice developed severe hydrocephalus, characterized by a domed head, by postnatal day (P)6 and markedly enlarged lateral ventricles (LVs) at both P0 and P6 (Fig. 1a,b and Supplementary Fig. 1a). Quantification showed an approximately 10-fold increase in LV area relative to control littermates at P6, indicating rapid progression of ventricular enlargement (Fig. 1b). Periventricular heterotopias (PH) are gray-matter nodules composed of ectopic neurons located along the walls of the lateral ventricles(32, 33). Human patients with CRB2 mutations frequently show PH in addition to hydrocephalus(34). Consistent with this, nodules of ectopic cells anchored to the ventricular wall were present in all CRB-dko^Nes^ brains examined and absent in controls (Fig. 1b’). NeuN staining at P6 revealed that NeuN^+^ neurons accounted for more than 40% of total cells within these nodules (Fig. 1c, e). A substantial fraction of cells was Cux1^+^ late-born neurons forming dense clusters, whereas CTIP2^+^ early born neurons represented only a minor fraction (Fig. 1d, 1f–g). These findings suggest that PH in CRB-dko^Nes^ mice arise predominantly from late-born cortical neurons, providing an experimental system to investigate their cellular origins and to model CRB2-associated human hydrocephalus.

### CRB-dko ^Nes^ mice develop rostral aqueductal stenosis with blocked CSF flow and rostro-caudal ECs loss.

Given that aqueductal stenosis is reported in a subset of patients with CRB2 mutations(9, 10, 34, 35), we next asked whether the developing cerebral aqueduct is structurally and functionally compromised in CRB-dko^Nes^ mice. Intracerebroventricular (ICV) Fast Green injections at P7 showed that in control littermates dye progressed through the third and fourth ventricles, whereas in CRB-dko^Nes^ mice the dye failed to transit through the aqueduct, indicating a functional flow obstruction (Fig. 1h). Consistent with this, at P6, the rostral aqueduct was narrowed and the Foxj1^+^/S100β^+^ ependymal lining cells was profoundly depleted (Fig. 1i). Notably, despite severe loss of Foxj1^+^/S100b^+^ ECs and Arl13b^+^/AC3^+^ cilia, the caudal aqueduct remained structurally open, suggesting regional differences in the timing of Cre activity and/or distinct structural integrity dependence on ECs (Fig. 1j,k). Rostral aqueductal stenosis was also observed in CRB-dko^Nes^ mice at P0 (Supplementary Fig. 1b). Together, these data indicate that Crb2 loss impairs ependymal-cell generation along the length of the aqueduct and causes rostral aqueductal stenosis. Thus, CRB-dko^Nes^ mice recapitulate key features of CRB2-associated hydrocephalus, ventriculomegaly, aqueductal stenosis, and PH and provide a tractable model to study ECs generation.

### Crb2 has a minimal role in EC maintenance and maturation.

Because Crb proteins remain robustly expressed in ECs and the ChP epithelium throughout life(20), we asked whether Crb1/2 are required for ependymal maturation or maintenance. ECs are born during neurogenesis, but maturation prolongs into postnatal ages in the mouse (Fig. 2a)(36, 37). To distinguish the function of Crb2 in maturation, and/or maintenance of ECs from EC generation defects, we deleted Crb2 using Foxj1-Cre(38), which recombines multiciliated epithelia, including CP and ependyma, after lineage specification (Fig. 2a). Immunostaining confirmed efficient loss of apical CRB2 in Foxj1^+^ epithelia (Supplementary Fig. 2a,**b,c**). Unlike Nestin-Cre mediated deletion, CRB-dko^Foxj1^ mice showed no aqueductal stenosis at P21 (Supplementary Fig. 2d,**e**) and no ventriculomegaly at P8 or P21 (Fig. 2b,g). The lateral ventricular wall remained continuously lined by Foxj1^+^/S100β^+^, Arl13b^+^/AC3^+^ ciliated ependyma, with no significant changes in number at P8, P21 (Fig. 2c–f, 2h–k), or P60 (Supplementary Fig. 2f-i). Interestingly, we found that Pan-Crb staining, which detects all three Crb proteins, is sustained in ChP epithelium despite known Cre-mediated deletion, suggesting that Crb3 is the dominant Crb isoform in this tissue or that compensatory upregulation of it occurs when Crb1 and 2 are depleted (Supplementary Fig. 2a). While ventriculomegaly can also arise from ChP hypersecretion, in addition to neural defects(9) and aqueductal stenosis, this model does not isolate Crb function in CP epithelium.

Together, our extensive analyses of CRB-dko^Foxj1^ mice at multiple time points suggest that Crb1/2 function in ependymal generation in neural progenitors, rather than in maturation or maintenance, is likely a primary driver of aqueductal stenosis and ventriculomegaly.

### Crb2 loss in cortical progenitors drives ventriculomegaly, ECs loss, and impairs CSF transit without evidence of aqueductal stenosis.

Consistent with these clinical cohorts, in which CRB2-associated hydrocephalus is dominated by ventriculomegaly (82%) and aqueductal stenosis is detected in only a minority of cases (~ 27%)(9, 34), emerging works suggest that additional mechanisms, such as neural progenitor defects, impaired proliferation(39, 40), and altered tissue mechanics(41), also contribute to ventricular enlargement. To directly test whether ventriculomegaly is caused by cortical progenitor defects, we conditionally deleted Crb1/Crb2 using Emx1-Cre, which recombines dorsal cortical progenitors beginning around E9.5(42) and does not target ventral midbrain/aqueduct lineages or the ChP. This strategy allowed us to investigate the impact of Crb2 loss in cortical progenitors, without evidence of direct involvement of the aqueduct or the ChP. Histological analysis revealed severe ventriculomegaly in CRB-dko^Emx1^ brains, evident at birth (P0) and progressing through postnatal stages P8, P21 (Fig. 3a,f and Supplementary Fig. 3a), demonstrating that Crb2 loss in cortical progenitors causes ventricular expansion. Along the lateral ventricular wall, loss of apical Pan-Crb signal confirmed efficient recombination in the dorsal cortex (Fig. 3c,d,h and Supplementary Fig. 3b). AJs (β-catenin labeling) were disrupted, and Foxj1^+^/S100β^+^ ECs were markedly reduced at P0 and remained depleted at P8 and P21 (Fig. 3c–e, 3h,i,k and Supplementary Fig. 3b,**c,e**). Consistent with this ECs loss, ciliated cells (AC3^+^/Arl13b^+^) were also significantly diminished at P8 and P21 (Fig. 3j,l, and Supplementary Fig. 3d,**f**). Collectively, these findings indicate that Crb2 is required in cortical progenitors for proper ECs generation, and that its loss leads to ependymal depletion and ventriculomegaly in the absence of aqueductal stenosis.

To determine whether impaired CSF flow underlies ventriculomegaly in CRB-dko^Emx1^ mice, we assessed both functional CSF transit and aqueduct structure (Fig. 3). Aqueductal stenosis is a common cause of obstructive hydrocephalus(10, 22), so we first injected Fast Green dye into the lateral ventricle at P8. In controls, dye rapidly progressed through the aqueduct, indicating normal CSF circulation (Fig. 3m). In contrast, CRB-dko^Emx1^ brains exhibited marked dye retention within the lateral ventricles, with limited distal distribution, consistent with impaired CSF transit through the aqueduct (Fig. 3m). We next examined whether this functional impairment reflected structural narrowing or collapse of the aqueduct. The entire rostro-caudal axis revealed a patent, β-catenin-lined aqueduct in both wild-type and CRB-dko^Emx1^ mice at P0 and P8, with continuous Foxj1^+^/S100β^+^, Arl13b^+^/AC3^+^ ciliated ependyma along its length, consistent with preserved AJ integrity (Fig. 3n–r and Supplementary Fig. 4). Crb expression was readily detected in the aqueductal epithelium. Thus, CSF flow is impaired CRB-dko^Emx1^ mice, despite an anatomically open and ciliated aqueduct, indicating that Crb2 deletion in cortical progenitors leads to CSF flow obstruction in the absence of structural aqueductal stenosis, and that ventriculomegaly arises from a mechanism independent of physical aqueduct blockage.

### Crb2 has a minimal role in cortical progenitor proliferation and neurogenesis.

Because altered CSF flow might reflect primary defects(39, 40) in cortical progenitor proliferation and/or self-renewal division mode, we next asked whether Crb2 loss is associated with defects in these parameters in the embryonic dorsal cortex. To determine whether early cortical progenitor defects contribute to the CRB-dko^Emx1^ phenotype, we assessed progenitor pool, proliferation, and division mode in the embryonic dorsal cortex (Fig. 4). At E14.5 and E16.5, CTIP2 and TBR2 immunostaining showed comparable numbers of early-born neurons and intermediate/basal progenitors between Controls and CRB-dko^Emx1^ (Fig. 4a–f). To assess S-fraction, defined as a fraction of apical radial glial cells (aRGs) in DNA replication, we performed a bromodeoxyuridine, (BrdU) injection, a thymidine analog, which incorporates during DNA replication. Brains were harvested 30 min later at E14.5 and E16.5 and we quantified the S-phase fraction (BrdU^+^Sox9^+^/Sox9^+^ cells), and the aRG progenitor population changes (Sox9^+^ cells). The Sox9^+^ aRG number (per frame) and S-fraction were not significantly different between Controls and CRB-dko^Emx1^ (Fig. 4g–m). Finally, we injected the timed-pregnant mice with BrdU and harvested the brains 24 hrs later to determine division mode (proliferative/self-renewal division fraction) by measuring the fraction of BrdU-labeled cells that retain Sox9. We observed no significant change in the proportion of cells remaining aRG among total BrdU^+^ S phase cells at the time of injection after one cell cycle (BrdU^+^Sox9^+^/BrdU^+^ cells) (Fig. 4n–p), indicating that there were no obvious defects in the self-renewing/proliferative division of aRG.

Additionally, to gain further insights into molecular changes explaining the failure to develop ECs or cellular defects, we conducted bulk RNA sequencing of the dorsal cortex at E14.5 (N = 4 Controls, N = 3 CRB-dko^Emx1^), (Supplementary Fig. 5a). This time point was chosen as no visible cellular defects had been observed. Analysis of bulk RNA-seq data revealed comparable expression levels of progenitor proliferation and differentiation genes in Control and CRB-dko^Emx1^ (Supplementary Fig. 5). Bulk RNA-seq of E14.5 dorsal cortex (N = 4 Controls, N = 3 CRB-dko^Emx1^), prior to overt cellular abnormalities, revealed highly similar transcriptomes between Control and CRB-dko^Emx1^ samples (Supplementary Fig. 5b). The top-ranked DEGs did not show clear genotype-driven changes (Supplementary Fig. 5b), and the volcano plot identified only three statistically significant genes among ~ 18,000 transcripts (Supplementary Fig. 5c). Crb2 showed the most robust reduction, consistent with efficient deletion, whereas Pop4 and Arfgef2 reached significance with modest effect sizes (∣log2FC∣ < 1). Together, these results indicate that global transcriptional programs, including broad proliferation and differentiation signatures, are largely preserved in dorsal cortex at E14.5, supporting the interpretation that CRB2-dependent defects likely arise later and/or through cell-type-restricted or post-transcriptional mechanisms that are not readily captured by bulk RNA-seq. Notably, Arfgef2 encodes a membrane trafficking regulator, making it a candidate for follow-up in the context of epithelial polarity/junctional maintenance(43), although its small fold change at E14.5 suggests any effect is subtle at this stage.

Together, these data indicate that Crb2 deletion in cortical progenitors does not detectably alter cortical progenitor proliferation, progenitor self-renewal or differentiation mode at these stages. Thus, despite a patent, ciliated aqueduct and largely normal progenitor output, the persistent Fast Green stagnation is consistent with altered ventricular-wall mechanics rather than a structural obstruction-driven etiology.

### Crb2 loss in cortical progenitors disrupts AJs and ventricular integrity.

Because CRB proteins organize apical polarity and coordinate AJs with the apical actomyosin cytoskeleton(20, 22, 39–41) (Fig. 5a,d), CRB2 loss is expected to weaken the junctional belt that mechanically supports the ventricular surface. Many congenital hydrocephalus genes also converge on these polarity/junction pathways (including CRB2 and associated factors such as MPDZ and CCDC88C), pointing to reduced apical cohesion and contractile tension as a shared mechanism. Thus, assessing AJ integrity in CRB-dko^Emx1^ tests whether early polarity disruption produces a mechanically weaker ventricular lining that could passively expand under pressure when tissue stiffness/viscoelasticity is reduced.

Given evidence that reduced tissue stiffness/viscoelastic cohesion can permit passive ventricular expansion(44) and that AJs contribute to mechanical stability at the ventricular surface (41), we next examined whether Crb2 loss compromises AJ integrity in the CRB-dko^Emx1^ cortex (Fig. 5). Coronal E14.5, E15.5 and E16.5 sections from CRB-dko^Emx1^ and littermate Controls were co-immunostained for AJ markers (N-cadherin and β-catenin), together with the polarity protein Pals1. At E14.5, no obvious differences were observed between Control and CRB-dko^Emx1^ cortices in NMIIb, N-cadherin, or β-catenin localization; however, Pals1 expression was reduced in CRB-dko^Emx1^ cortices (Supplementary Fig. 6a-c). At E15.5, β-catenin, α-catenin, and N-cadherin distribution remained comparable between Control and CRB-dko^Emx1^ mice, whereas CRB and Pals1 were absent in CRB-dko^Emx1^ cortices (Supplementary Fig. 6d-f). At E16.5, no overt differences in overall brain morphology were detected between Control and CRB-dko^Emx1^ embryos (Fig. 5b). In Controls, AJ labeled with N-cadherin/β-catenin and co-aligned with Pals1 along the ventricular surface, whereas in CRB-dko^Emx1^ the AJ appeared discontinuous and Pals1 was substantially reduced (Fig. 5c). Because AJ continuity relies on actomyosin-generated tension and Crb2 has been linked to the regulation of Rho-ROCK-myosin activity(30, 45), we next examined apical actomyosin organization (Fig. 5d). Consistent with reduced apical contractility, reduced phosphorylated myosin regulatory light chain (pMLC, the active form of MLC that promotes myosin II motor activity) and non-muscle myosin IIb (NMIIb, the myosin II heavy-chain isoform enriched apically) were diminished in CRB-dko^Emx1^ (Fig. 5e,f). In addition, phalloidin labeling revealed disruption of the apical F-actin ring at ventricular endfeet in CRB-dko^Emx1^ (Fig. 5g–i).

Thus, Crb2 may contribute to establishing a cohesive, mechanically robust AJ network at the ventricular surface. In its absence, the ventricular lining appears less cohesive and may be less able to withstand intraventricular pressure. Together with the impaired CSF transit observed in CRB-dko^Emx1^ mice (Fig. 3), these findings suggest that polarity/junctional disruption is likely associated with altered ventricular-wall mechanics and may contribute to ventricular enlargement.

### Crb2 loss in cortical progenitors is associated with early ventricular lining cell detachment and increased CSF access to periventricular tissue.

Ventriculomegaly can arise not only from obstructed CSF flow but also from structural weakening of the ventricular lining(44, 46, 47), which may permit ventricular expansion and increased CSF access to adjacent brain tissue. In such cases, disruption of the ependymal/ventricular barrier may represent a key pathogenic step. Since ventricular lining disruption is a common feature of hydrocephalus, we asked whether Crb2 loss causes early lining cell detachment and greater CSF access to the periventricular cortex.

To assess whether ventricular lining progenitors detach from the ventricular surface in association with AJ disruption, we selectively labeled ventricular lining cells at E17.5 using FlashTag, an in vivo pulse-labeling method based on ICV injection of a cell-permeable fluorescent dye (CFSE)(48), and collected brains at E18.5. In Controls, FlashTag^+^ labeled cells formed a continuous apical band, but in CRB-dko^Emx1^, a subset protruded into the lumen or appeared as free-floating clusters, consistent with detachment of ventricular lining cells (Fig. 6a,b). Because weakening of the ventricular lining may allow increased access of CSF to adjacent brain tissue, we next tested whether ventricular lining/junctional disruption was accompanied by increased permeability to ventricular contents. To test this, we injected biotinylated dextran amine (BDA) into the lateral ventricle (LV) at P8 and collected brains 20 min later to assess barrier function by examining the entry of biotinylated dextran(49). We visualized the ventricular surface in thick vibratome sections using high-resolution confocal imaging of serial z-stacks. Three-dimensional reconstruction and rendering of these image stacks with Imaris then revealed the spatial organization and deformation of the lumen and ventricular lining at the whole-structure level, rather than in isolated single sections. In Control mice, streptavidin signal was not detected in the cortex or along the ependymal surface, whereas in CRB-dko^Emx1^ brains signal was detected in the periventricular parenchyma, including labeling of nearby cell bodies and processes (Fig. 6c). To ensure accurate intracerebroventricular (ICV) delivery, FlashTag was co-injected in each condition, enabling visual verification through robust GFP labeling in choroid plexus and ventricular lining cells.

These findings indicate increased BDA penetration into adjacent cortical tissue in CRB-dko^Emx1^ and are consistent with the ventricular lining/junctional abnormalities observed in CRB-dko^Emx1^. Overall, these data indicate that Crb2 loss is accompanied by early ventricular lining disruption and increased permeability of ventricular contents into the adjacent periventricular tissue.

### Acute bumetanide treatment attenuates ventriculomegaly in CRB-dko ^Emx1^ mice without rescuing ependymal integrity.

In high-risk congenital hydrocephalus, many of the newly identified risk genes disrupt neurogenesis and cortical development, producing structurally weakened brain tissue that develops ventriculomegaly even when CSF pathways are patent and surgical CSF diversion is performed, indicating that altered brain development alone can be sufficient to drive ventricular expansion(39, 40, 50). In contrast, our CRB-dko^Emx1^ model does not exhibit primary neurogenesis defects, but rather junctional and mechanical abnormalities of the ventricular wall, and recent work indicates that ventriculomegaly can arise not only from obstructed CSF flow or neurogenesis defects but also from altered tissue mechanics and increased CSF load on the ventricular wall(50, 51). Because Crb-mediated ventriculomegaly in CRB-dko^Emx1^ mice reflects reduced tissue mechanical tension (reduced NMIIb, pMLC) secondary to impaired junctional integrity, rather than progenitor proliferation defects, we hypothesized that ventricular size in this context should remain sensitive to CSF load. We therefore treated CRB-dko^Emx1^ pups at P0 with bumetanide, an inhibitor of the sodium (Na+), potassium (K+), and chloride (Cl−) cotransporter (NKCC1) that reduces CSF production(52) (Fig. 6d). Again, FlashTag was co-injected to enable visual confirmation of accurate ICV delivery in every group.

At P8, H&E staining revealed that bumetanide-injected CRB-dko^Emx1^ mice exhibited markedly smaller lateral ventricles than vehicle-injected CRB-dko^Emx1^ littermates (Fig. 6e,f). Ventricular area quantification confirmed a significant reduction, bringing ventricular size in bumetanide-injected CRB-dko^Emx1^ mice at P8, close to that of P0 CRB-dko^Emx1^ mice, indicating that the progression of ventriculomegaly can be halted when CSF load is acutely reduced (Fig. 6g,h). In contrast, analysis of the ventricular lining showed that bumetanide failed to restore ependymal organization: FlashTag labeling and Foxj1 immunostaining demonstrated persistent disruption of the ECs layer in CRB-dko^Emx1^ mice, and Pan-Crb apical staining remained absent in the dorsal cortex, consistent with sustained loss of apical polarity (Fig. 6i and Supplementary Fig. 7).

### Crb2 loss in the midbrain disrupts aqueduct integrity but does not prevent the generation of multiciliated ependymal cells.

Because human CRB2 mutant cases exhibit aqueductal loss and ependymal rosettes while retaining ECs, it is likely that ECs can be generated in the absence of Crbs(22). Based on our findings in cortical progenitors, we reasoned that loss of Crbs disrupts AJs(16, 53–58) leading to detachment of ventricular-surface cells, which are subsequently displaced by CSF flow rather than remaining locally in Crb mutant animals. We hypothesized that early caudal aqueduct closure could transiently trap detached cells in the rostral region. To this end, we deleted Crb1/2 in the midbrain using Wnt1-Cre2 (CRB-dko^Wnt1-Cre2^)(59) and analyzed aqueduct development during late embryogenesis. H&E analysis at E17.5 revealed caudal aqueduct closure in mutants, whereas the rostral aqueduct remained patent (Supplementary Fig. 8a), providing a developmental context to assess whether ventricular lining cells could become retained or mislocalized within the disrupted aqueduct. Consistent with Crb1/2 loss, Pan-Crb staining was absent from both the rostral and caudal aqueduct, and AJ disruptions (N-cadherin labeling) were observed in both rostral and caudal aqueduct in CRB-dko^Wnt1-Cre2^ mice (Supplementary Fig. 8b,**c**).

Postnatally, CRB-dko^Wnt1-Cre2^ mice developed hydrocephalus with impaired CSF flow to the 4th ventricle, indicative of aqueductal stenosis (Fig. 7b,c and Supplementary Fig. 9a). When we examined the aqueduct, the rostral aqueduct failed to form in the Crb mutant, and in the caudal region, abnormal cell accumulation was observed within the closed aqueduct area at both P0 and P7 (Fig. 7d and Supplementary Fig. 9b,**c**). First, we identified that the cells are Crb deficient, as Pan-Crb immunostaining is absent, unlike the strong expression in the ventricular lining in Controls in both the rostral and caudal aqueduct (Fig. 7e and Supplementary Fig. 8c). To detect whether cells that have accumulated caudally are multiciliated ECs, we examined the presence of mature ECs marked by FoxJ1^+^/S100β^+^ and Arl13b^+^/AC3^+^. Unlike the rostral aqueduct, abundant multiciliated ECs are detected in the caudal aqueduct, suggesting these ECs are developed from either cells detached from the rostral ventricular lining or locally retained ECs at both P0 and P7 (Fig. 7f,g,i,j, and Supplementary Fig. 9f,**h**).

To gain insights about the potential rostral origin of these cells, we examined subcommissural organ (SCO) cells, which are specifically located in the rostral aqueduct and produce glycoprotein-rich Reissner’s fiber. While AFRU^+^ SCO-associated cells and fibers are restricted in the rostral aqueduct in control, in Crb mutants, the AFRU signal is undetectable in the rostral side but accumulated ectopically in the caudal aqueduct region at P0 and P7 (Fig. 7h,k and Supplementary Fig. 9e, **g**). This supports the idea that ectopically accumulated ECs in the caudal region include detached rostral-born ECs.

Overall, these data show that Crb2 loss does not impair ECs generation but instead disrupts ventricular integrity, causing detachment and rostro-caudal displacement of ventricular lining cells, including ependymal and SCO cells.

## Discussion

Our study identifies disruption of Crb2 function in neural progenitors as the major driver of hydrocephalus in Crb2 mutants. By deleting Crb1 and Crb2 in four distinct lineages, pan-neural (Nestin-Cre), mid and hindbrain (Wnt1-Cre2), cortical (Emx1-Cre), and multiciliated epithelia (Foxj1-Cre), we show that Crb2 acts in neural progenitors, not in differentiated ependyma or CP, to preserve the structural and mechanical integrity of the ventricular system. Loss of Crb2 in neural progenitors causes a primary defect in AJs that results in a mechanically weakened, leaky ventricular lining in cortical mutants. Deleting Crb1/2 in already lineage-specified multiciliated cells leaves ependymal architecture intact into adulthood, while midbrain Crb2 loss disrupts aqueduct patency yet permits EC and SCO-cell generation, with detached rostral-born cells accumulating caudally within the stenosed aqueduct. Acute bumetanide treatment at P0 attenuates ventriculomegaly at P8 in CRB-dko^Emx1^ mice by reducing CSF load without restoring ECs integrity, indicating that ventricular expansion remains highly sensitive to CSF pressure even when the ventricular wall is structurally compromised. Together, these data reframe CRB2-associated hydrocephalus as a progenitor-stage adhesion disorder, with downstream ventricular expansion remaining sensitive to CSF load.

Our results with Crb2 deficiency in neural progenitors provide new insight into how Crb2 safeguards, rather than molecularly specifies, ependymal precursors. ECs arise from aRG during neurogenesis, but their distribution and fate during this period are difficult to track because reliable EC markers such as Foxj1 are not robustly expressed in cortical ventricular lining cells until around P0. In CRB-dko^Emx1^ mutants, most FlashTag-labeled ventricular lining cells are detaching or poised to detach from the ventricular surface by E18.5, when cortical neurogenesis is complete, indicating that these displaced cells likely include ependymal precursors. Consistent with this idea, our CRB-dko^Wnt1-Cre2^ experiments show that Crb2 loss can disrupt aqueduct integrity while still permitting generation and caudal accumulation of multiciliated ECs and SCO-derived cells, supporting a model in which Crb2 maintains ventricular wall cohesion and positioning of EC precursors.

Our results suggest that Crb2 functions to prevent loss of ventricular lining cells by providing AJ-mediated integrity, rather than playing an instructive role in their fate. Consistent with this, a human postmortem study of a CRB2 mutation associated with aqueduct atresia reported preserved ECs(22). In line with this, Crb1/2-dko^Wnt1-Cre2^ mice retain ECs with multiple cilia in the caudal aqueduct despite loss of Crb1/2, providing compelling evidence that the molecular program for EC fate is not apparently affected. This differs from zebrafish studies linking Crb2 homolog function to differentiation and the motility of cilia in renal cilia, suggesting that Crb-dependent regulation of multiciliated cells may be context- or species-dependent(60). In summary, these data support the idea that Crb2 primarily protects ventricular lining cells, including ependymal precursors, from being lost, but does not directly control their fate or lineage progression.

Our study demonstrates that Crb2-dependent polarity and adhesion in neural progenitors support ventricular surface integrity and normal ventricular lining development, and that Crb2 loss in these functions is associated with ventriculomegaly. CRB proteins are implicated in many cellular processes, including apical domain specification, cell adhesion, AJ formation, signaling regulation, and ciliogenesis. Our study suggests that the major Crb2 function in cortical progenitors is AJ establishment/and stability during late neurogenesis. AJ formation requires junctional actomyosin activity, which promotes cadherin clustering(61). Crb2 has been implicated in regulating actomyosin activity through Rho-ROCK-myosin signaling. However, prior studies report conflicting models; some suggest that CRB2 inhibits Rho-ROCK activity via aPKC-dependent regulation, whereas others propose that CRB2 promotes Rho-ROCK signaling by recruiting RhoGEF in *Drosophila*(30). Consistent with the latter, our analyses show reduced phosphorylated myosin light chain (pMLC) in Crb2 mutants, supporting a role for Crb2 in promoting apical actomyosin activity at the junction. Because we observed that Pals1, an interacting polarity complex protein, is diminished earlier, before the junction disruption, whereas decreased active myosin correlates with junction defects, this suggests that loss of active myosin in the absence of Crb1/2 may be the major contributor to AJ instability.

It is notable that junction defects in cortical progenitor-specific Crb1/2 mutants are detectable when neurogenesis is nearly complete in CRB-dko^Emx1-Cre^. As we have not observed progenitor reduction in number or alterations in self-renewal/proliferative division mode, progenitor proliferation defects are minimal in Crb1/2 mutants. Accordingly, we did not observe any molecular changes related to proliferation in RNA-seq experiments prior to junction disruption. This is consistent with the absence of neurogenesis defects when the aPKCl, another core apical polarity component, is deleted using Nestin-Cre, despite clear junction defects in later stages, such as E15.5(62). Therefore, together with previous studies, our study provides evidence that AJ loss later in neurogenesis does not affect neurogenesis. Given the potential functional redundancy between Crbs and aPKCl, it would be interesting to remove both and determine whether the loss of AJC can be achieved earlier and whether this early loss affects neurogenesis.

Importantly, the absence of apparent neurogenesis defects in Crb2 mutants clarifies the role of cell junction defects in the compromised biomechanical properties underlying ventriculomegaly, without the confounding effects of aberrant neurogenesis. A group of genes that cause congenital hydrocephalus affects cortical neurogenesis by modulating mitogenic/growth signaling pathways, such as Shh and mTOR(63). Consequently, some of these are comorbid with other neurodevelopmental disorders, such as autism spectrum disorders or microcephaly (64). Contrary to these models, in which CSF diversion is ineffective at reducing ventriculomegaly, the significant reduction in ventricular size after bumetanide treatment in Crb mutants supports a distinct pathogenesis. Because junctional integrity provides biomechanical rigidity, the disruption of junctional integrity correlates with the ventriculomegaly due to hypercompliance of the ventricular wall and loss of barrier function of the ventricular lining. Our results, showing neuronal labeling only in Crb mutants after BDA injected into the ventricle, also provide evidence of compromised barrier function. It would be interesting in future studies to increase actomyosin activity using pharmacological or genetic approaches to restore junctional integrity and barrier function, thereby reducing consequent ventriculomegaly for future investigation.

Our use of different Cre drivers to assess cell-type-specific function of Crb1/2 are painstaking; however, they have yielded important clarity into relevant cell types and developmental timing in hydrocephalus and PH. Because Nestin-Cre targets most neural cells in the CNS, the human phenotype can be faithfully recapitulated. PH is observed only with Nestin-Cre but not with Emx1-Cre, demonstrating the potential non-cell-autonomous effects of Crb1/2 on neural progenitors. As Nestin-Cre targets progenitors in other brain regions, such as the developing aqueduct. We propose that aqueduct stenosis-mediated pressure on cortical aRG may play an important role in heterotopia formation. Because the degree and timing of ventriculomegaly are greater and occur earlier when aqueduct stenosis is present, increased ventricular tension may facilitate earlier disruption of the AJ. This AJ disruption during mid-neurogenesis or stronger degree in late neurogenesis may be required for PH formation, as seen in junction or cytoskeletal regulator mutants with the condition(32, 65–67). Further experiments to increase pressure in Crb1/2 dko^Emx1-Cre^ mice by physically blocking the aqueduct or increasing CSF volume during neurogenesis may provide evidence for this proposition.

Interestingly, we observed differential expressions of Crb family proteins in ECs versus ChP epithelial cells. Crb2 is the major Crb protein in mammals, expressed in many epithelial cells, especially in the early embryo, whereas CRB1’s function is mainly limited to the retina. Accordingly, Crb2 knockout results in early embryonic lethality due to gastrulation defects (19, 68, 69). Crb3, which lacks most of its extracellular domain mediating adhesive cellular interactions, is neonatally lethal when knocked out(70). Our pan-Crb immunostaining revealed that Crb1/2 dko^foxj1^ eliminates pan-Crb immune positivity in ECs almost completely, but ChP epithelium retains a Crb immune-positive signal. This result indicates that Crb3 may be the major Crb family protein expressed in the ChP or may be compensatory upregulation when other Crb proteins are absent. Because a previous study showed that a strong Crb3-specific signal in the ChP in the absence of Crb1/2 deletion, this supports the idea that Crb3 is the major Crb protein in the ChP, and that the deletion of Crb1/2 may not cause any severe defects in the ChP(67, 71, 72). The study of the CRB-polarity complex protein, MPDZ, demonstrates the importance of defects in ChP epithelium permeability and their potential role in hydrocephalus pathogenesis(20, 23). As Crb1/2 are dispensable in the ChP and cortical progenitor-specific Crb1/2 deletion is sufficient to generate ventriculomegaly, the contribution of ChP defects, such as hypersecretion or permeability changes, to hydrocephalus can be excluded in our model.

Our study may suggest bumetanide(52) as a therapeutic candidate for CH with a compromised biomechanical barrier(73, 74). Accumulating evidence indicates that ChP NKCC1-dependent transport can drive pathological CSF hypersecretion in acquired hydrocephalus models, and that inhibiting this pathway can reduce CSF secretion and ventriculomegaly(75). Accordingly, we used bumetanide with the goal of dampening CSF secretion in our model by directly targeting this ChP transport pathway. Because NKCC1 is positioned on the apical (ventricular-facing) membrane of ChP epithelium and systemic bumetanide has limited CNS exposure, direct delivery into the CSF, as in our ICV approach, is expected to improve target engagement while avoiding systemic tolerability limits that have hampered older “CSF-reducing” diuretic strategies(76). A single bumetanide injection reduced CSF secretion, significantly decreased ventriculomegaly, and prevented further enlargement, reinforcing the critical importance of reducing the biomechanical pressure. Together, these findings indicate that CSF-driven forces are sufficient to modulate the degree of ventriculomegaly in the setting of a mechanically compromised ventricular wall, but that enduring epithelial and junctional defects impose a structural limit on recovery, underscoring the need for therapeutic strategies that jointly normalize CSF dynamics and repair ventricular wall integrity.

## Conclusions

Crb2-dependent polarity and adhesion in neural progenitors emerge as a key safeguard of ventricular wall integrity, preventing junctional failure, loss of the ventricular lining, and abnormal CSF access to periventricular tissue in congenital hydrocephalus. By disentangling progenitor-stage adhesion defects from ependymal fate and aqueduct patency and showing that ventricular size remains highly sensitive to CSF load when the wall is mechanically weakened, this work reframes CRB2-associated hydrocephalus as a disorder of barrier integrity and highlights ventricular biomechanics and brain fluid dynamics as promising targets for future therapies.

## Supplementary Material

This is a list of supplementary files associated with this preprint. Click to download.

• SupplementaryMaterial1Fig1.pdf

• SupplementaryMaterial2Fig2.pdf

• SupplementaryMaterial3Fig3.pdf

• SupplementaryMaterial4Fig4.pdf

• SupplementaryMaterial5Fig5.pdf

• SupplementaryMaterial6Fig6.pdf

• SupplementaryMaterial7Fig7.pdf

• SupplementaryMaterial8Fig8.pdf

• SupplementaryMaterial9Fig9.pdf

• Additionalfile10.zip

• SupplementaryInformation.docx

## Figures and Tables

**Figure 1:**
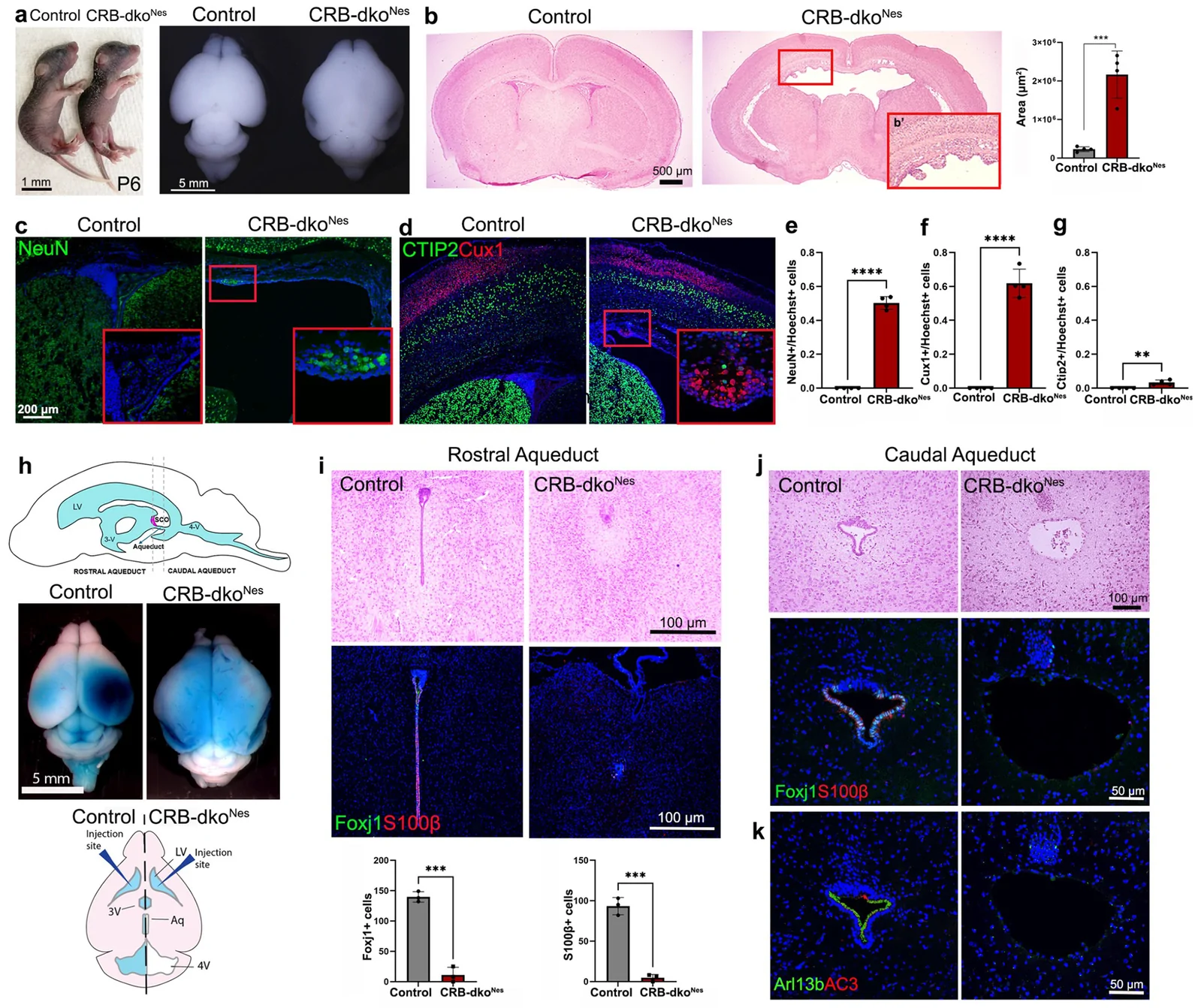
Crb2 loss in neural progenitors causes hydrocephalus, periventricular heterotopia (PH), and aqueductal stenosis. **(a)** Representative images of Control and CRB-dko^Nes^ pups at P6 showing domed shaped head morphology and dorsal views of dissected brains, N=3 Controls, N=3 CRB-dko^Nes^. **(b)** Representative coronal H&E-stained sections and quantification showing enlarged lateral ventricles (LV) in P6 CRB-dko^Nes^ mice compared with Controls; N=4 Controls, N=4 CRB-dko^Nes^; P = 0.0007. **(b’)** Red box indicates magnified regions of PH. **(c)** Immunostaining for NeuN labeling PH in CRB-dko^Nes^ brains, with quantification of NeuN^+^ cells within PH, N=4 Controls, N=4 CRB-dko^Nes^. Red boxes indicate magnified regions of PH. **(d)** Immunostaining for CTIP2 (early born neurons, green) and Cux1 (late-born neurons, red) with **(e-g)** corresponding cell-count quantifications, N=4 Controls, N=4 CRB-dko^Nes^; (g) P = 0.0030. **(h)** Schematic showing rostral and caudal aqueduct levels analyzed, intracerebroventricular (ICV) Fast Green injection strategy, and expected cerebrospinal fluid (CSF) flow path. Whole-brain images after Fast Green injection at P7 show impaired CSF flow through the aqueduct in CRB-dko^Nes^ mice. N=3 Controls, N=3 CRB-dko^Nes^. **(i)** Representative rostral aqueduct H&E-stained sections and representative immunostaining for Foxj1 (green) and S100β (red) in the rostral aqueduct. Quantification of Foxj1^+^ and S100β^+^ ependymal cells at the rostral aqueduct. N=3 Controls, N=3 CRB-dko^Nes^, P = 0.0001 (left), P = 0.0002 (right). **(j)** Representative H&E-stained coronal sections at the caudal aqueduct level at P6. Representative image of caudal aqueduct for Foxj1/S100β immunostaining and **(k)** representative images of caudal aqueduct for Arl13b (cilia) and AC3 immunostaining. N=3 Controls, N=3 CRB-dko^Nes^. (b,e,f,g,i): Two-tailed unpaired t-test was performed for statistical significance. In the plots significance is indicated as **P<0.01, ***P< 0.001, ****P < 0.0001. Error bars represent mean ± SD, points represent individual animals.

**Figure 2:**
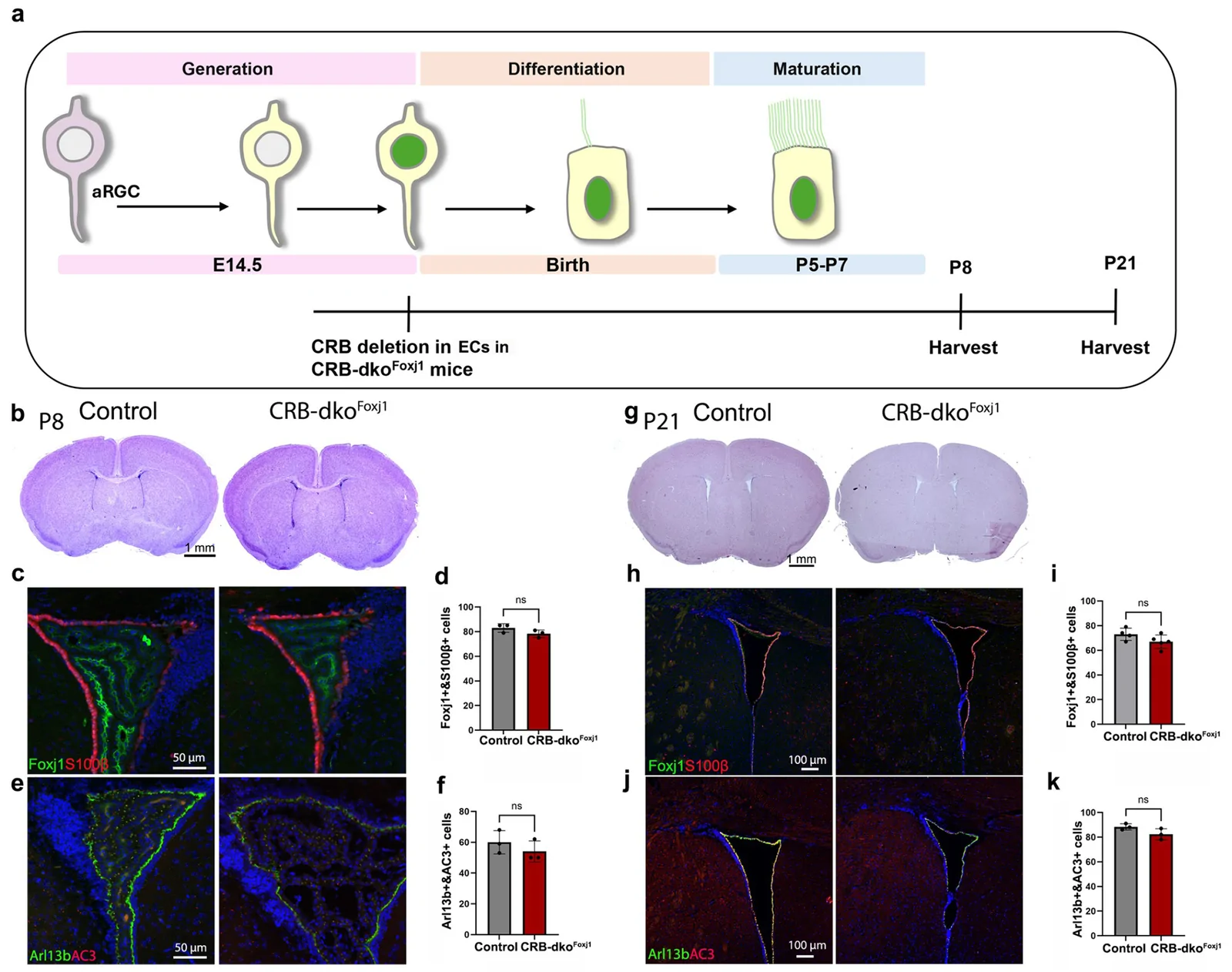
The Crb2 role in ependymal cell (EC) maintenance and maturation is minimal. **(a)** Schematic showing the timeline of ependymal cell generation, differentiation, and maturation, with Foxj1-Cre-mediated CRB deletion in CRB-dko^Foxj1^ mice and brain harvest timepoints indicated. **(b)** Representative H&E-stained coronal sections at P8 from Control and CRB-dko^Foxj1^ mice showing the Lateral Ventricles (LV) of the brain. **(c)** Representative immunostainings of the lateral ventricles (LV) of the brains at P8 stained for Foxj1 (ependymal lineage marker, green) S100β (mature ependymal marker, red) and **(d)** quantification at P8 of Foxj1^+^/S100β^+^ cells per section. N=3 Controls, N=3 CRB-dko^Foxj1^, P = 0.1550. **(e)** Representative immunostaining for AC3 (ciliary marker, red) and Arl13b (ciliary marker, green) and **(f)** quantification of AC3^+^/Arl13b^+^ ciliated cells per section. N=3 Controls, N=3 CRB-dko^Foxj1^, P = 0.3679. **(g)** Representative H&E-stained coronal sections at P21 from Controls and CRB-dko^Foxj1^. **(h)** Representative immunostaining at P21 for Foxj1/S100β and **(i)** quantification of Foxj1^+^&S100β^+^ cells per section. N=4 Controls, N=5 CRB-dko^Foxj1^, P = 0.1314. **(j)** Representative immunostaining at P21 for AC3/Arl13b and **(k)** quantification of Arl13b^+^&AC3^+^ ciliated cells. N=3 Controls, N=3 CRB-dko^Foxj1^, P = 0.1145. (d,f,i,k): Two-tailed unpaired t-test, ns, not significant. Bars show mean ± SD, points represent individual animals.

**Figure 3:**
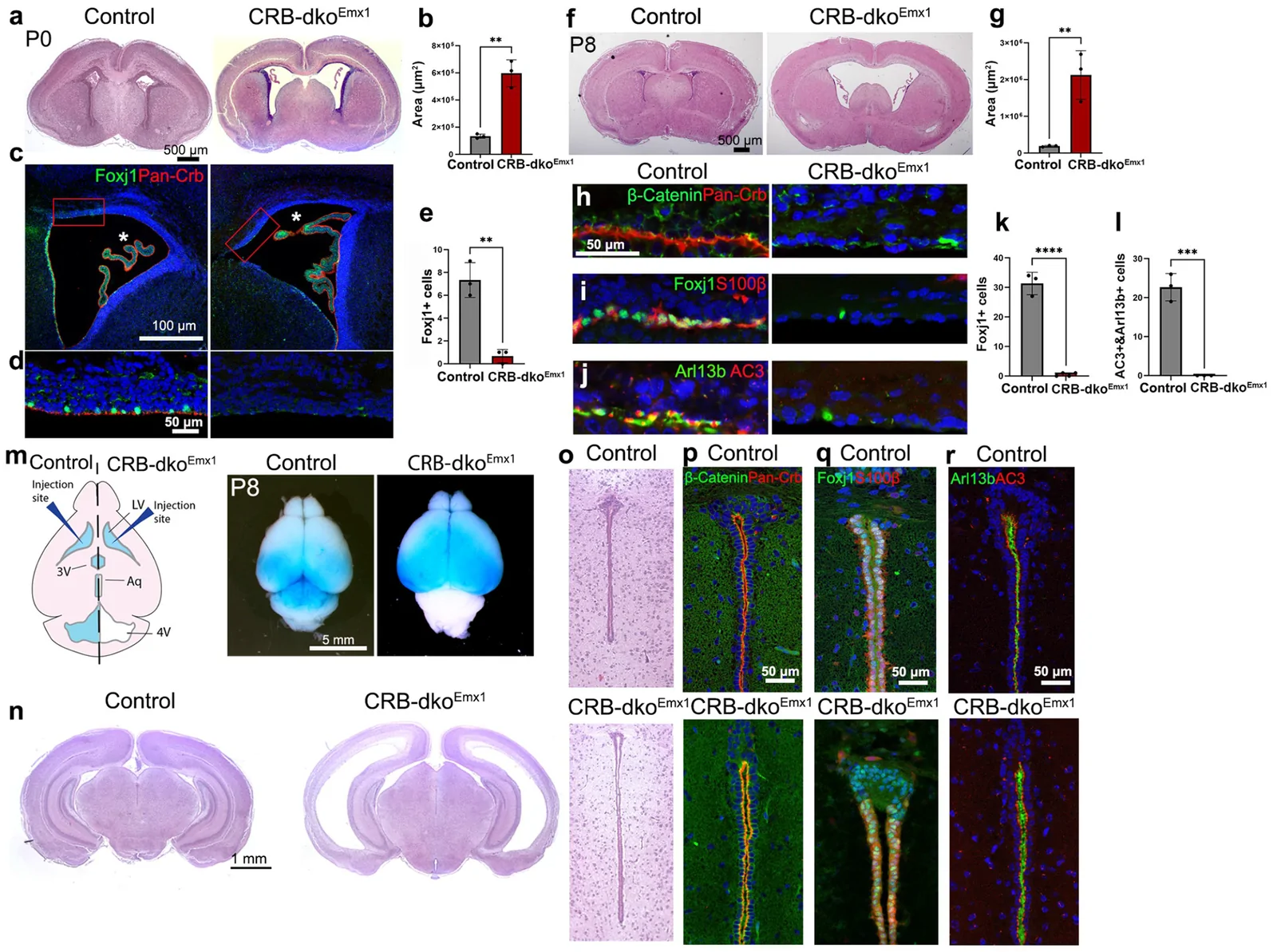
Crb2 loss in cortical progenitors drives ventriculomegaly, ependymal cells loss (ECs) and impairs CSF transit without evidence of aqueductal stenosis. **(a)** Representative H&E-stained coronal sections from Control and CRB-dko^Emx1^ mice at postnatal day 0 (P0) and **(b)** quantification of ventricular area; P = 0.0013. **(c)** Representative immunostainings of the ventricular wall at P0 stained for Foxj1 (ependymal lineage marker, green) and pan-Crb (red). (*) indicate pan-Crb staining in choroid plexus. **(d)** Higher-magnification views of the ventricular lining showing reduced FoxJ1^+^ cells in CRB-dko^Emx1^ and loss of Crb2 signal confirming efficient deletion and **(e)** quantification of Foxj1^+^ cells along the ventricular lining at P0. N=3 Controls, N=3 CRB-dko^Emx1^, P = 0.0021. **(f)** Representative H&E-stained coronal sections at P8 showing enlarged ventricles in CRB-dko^Emx1^ and **(g)** quantification of ventricular area at P8, N=3 Controls, N=3 CRB-dko^Emx1^, P = 0.0071. **(h)** Representative immunostaining at P8 showing junctional β-catenin (green) with Pan-Crb (red, top), **(i)** Foxj1 (green) with S100β (mature ependymal cells marker, red, middle), and **(j)** the ciliary marker Arl13b (green) with S100β (red, bottom), nuclei (Hoechst, blue). **(k)** Quantification of Foxj1^+^ cells per section (N=3 Controls, N=4 CRB-dko^Emx1^) and **(l)** AC3^+^&Arl13b^+^ ciliated cells per section at P8 (N=3 Controls, N=3 CRB-dko^Emx1^), P = 0.0004. **(m)** Schematic showing intracerebroventricular (ICV) Fast Green injection strategy and expected cerebrospinal fluid (CSF) flow path. Dorsal views at P8 brains showing distribution of Fast Green dye after ICV injection (Controls vs CRB-dko^Emx1^), N=6 Control, N=7 CRB-dko^Emx1^. **(n)** Representative H&E-stained coronal sections of the rostral aqueduct. **(o)** Higher-magnification views of the H&E-stained coronal sections of the rostral aqueduct in Control vs CRB-dko^Emx1^ mice. **(p)** Immunostainings at the rostral aqueduct for β-catenin/pan-Crb, **(q)** Foxj1^+^/S100β^+^, and **(r)** Arl13b^+^/AC3^+^ cells to visualize ependymal lining and cilia. (l): N=3 Controls, N=3 CRB-dko^Emx1^, (m): N=3 Controls, N=3 CRB-dko^Emx1^, (n): N=3 Controls, N=4 CRB-dko^Emx1^. (b,e,g,k,l): Two-tailed unpaired t-test was performed for statistical significance. In the plots significance is indicated as **P<0.01, ***P< 0.001, ****P < 0.0001. Error bars represent mean ± SD, points represent individual animals.

**Figure 4:**
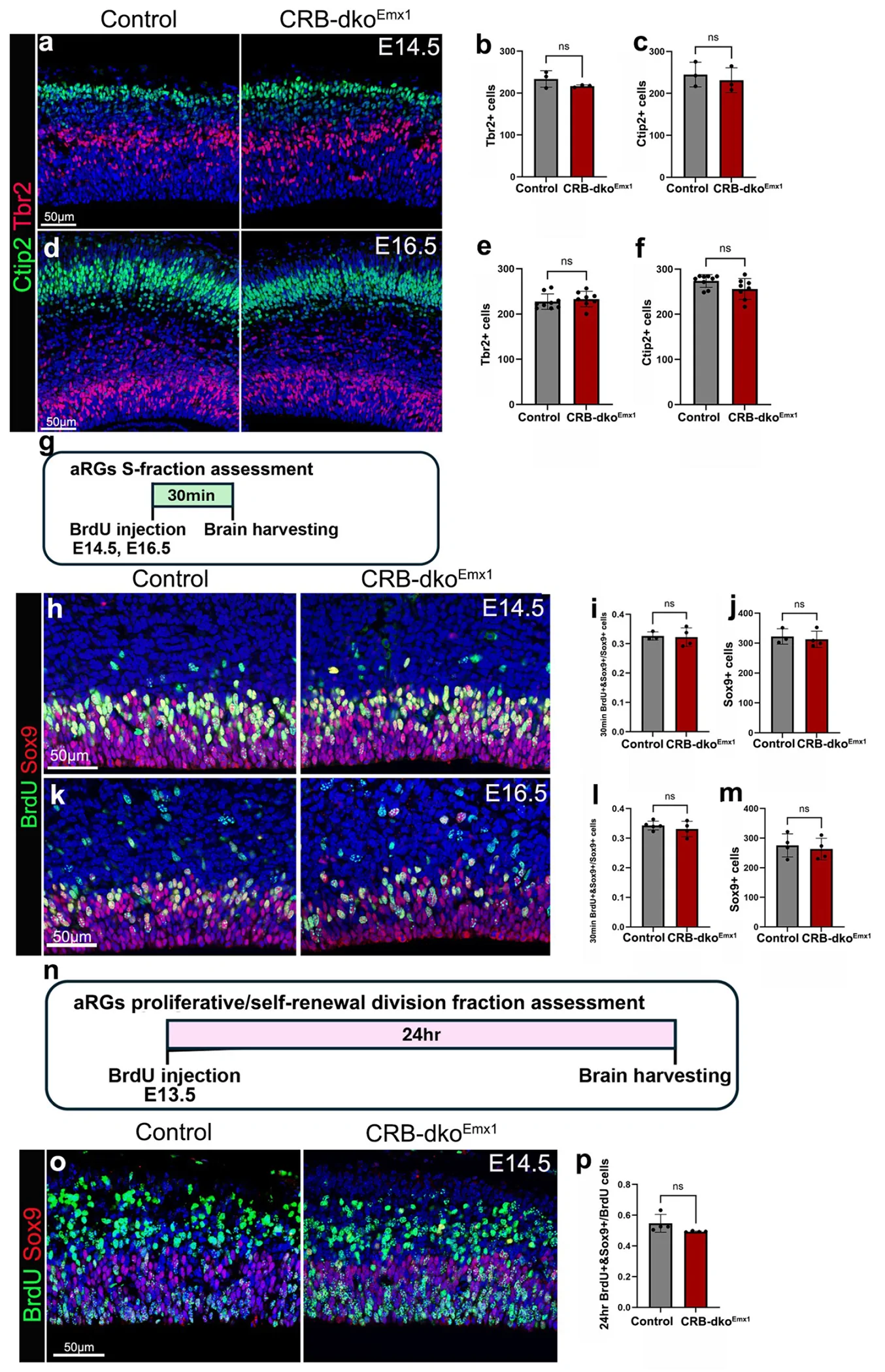
Crb2 has a minimal role in cortical progenitor proliferation and neurogenesis. **(a)** Representative immunostainings of coronal dorsal cortical sections at E14.5 labeled for CTIP2 (early born neurons) and TBR2 (intermediate/basal progenitors). **(b)** Quantification of Tbr2^+^ cells per section, P = 0.2081 and **(c)** CTIP2^+^ cells per section (N=3 Controls, N=3 CRB-dko^Emx1^), P = 0.6051. **(d)** Representative immunostainings of coronal dorsal cortical sections for CTIP2 (early born neurons) and TBR2 (intermediate/basal progenitors) at E16.5. **(e)** Quantification of Tbr2^+^ cells per section, P = 0.5075 and **(f)** CTIP2^+^ cells per section (N= 9 Controls, N=8 CRB-dko^Emx1^), P = 0.0717. **(g)** Schematic of aRG S-phase fraction assay. **(h)** Representative immunostainings of coronal dorsal cortical sections at E14.5, labeled for BrdU (S-phase fraction) and Sox9 (marker for aRGCs) at 30min, (Controls vs CRB-dko^Emx1^). **(i)** Quantification of S-phase fraction (30 min BrdU^+^&Sox9^+^/Sox9^+^ cells) at E14.5, P = 0.8470 and **(j)** quantification of progenitors’ (aRGCs) population (Sox9^+^ cells) at E14.5, N=3 Control, N=4 CRB-dko^Emx1^, P = 0.6633. **(k)** Representative immunostainings of coronal dorsal cortical sections at E16.5, labeled for BrdU (S-phase fraction) and Sox9 (marker for aRG) at 30min. **(l)** Quantification of S-phase fraction (30min BrdU^+^&Sox9^+^/Sox9^+^ cells) at E16.5, N=5 Controls, N=4 CRB-dko^Emx1^, P = 0.4225 and **(m)** quantification of progenitors’ (aRGCs) population (Sox9^+^ cells) at E16.5, N=4 Controls, N=4 CRB-dko^Emx1^, P = 0.6564. **(n)** Schematic of aRG proliferative/self-renewal division fraction assay. **(o)** Representative immunostainings of coronal dorsal cortical sections at E14.5, labeled for BrdU (S-phase fraction) and Sox9 (marker for aRGCs) at 24hrs. **(p)** Quantification of proliferative/self-renewal division fraction (24hr BrdU^+^&Sox9^+^/BrdU^+^ cells) at E14.5, P = 0.1131. (b,c,e,f,i,j,l,m,p): Two-tailed unpaired t-test, ns, not significant. Bars show mean ± SD, points, individual animals.

**Figure 5:**
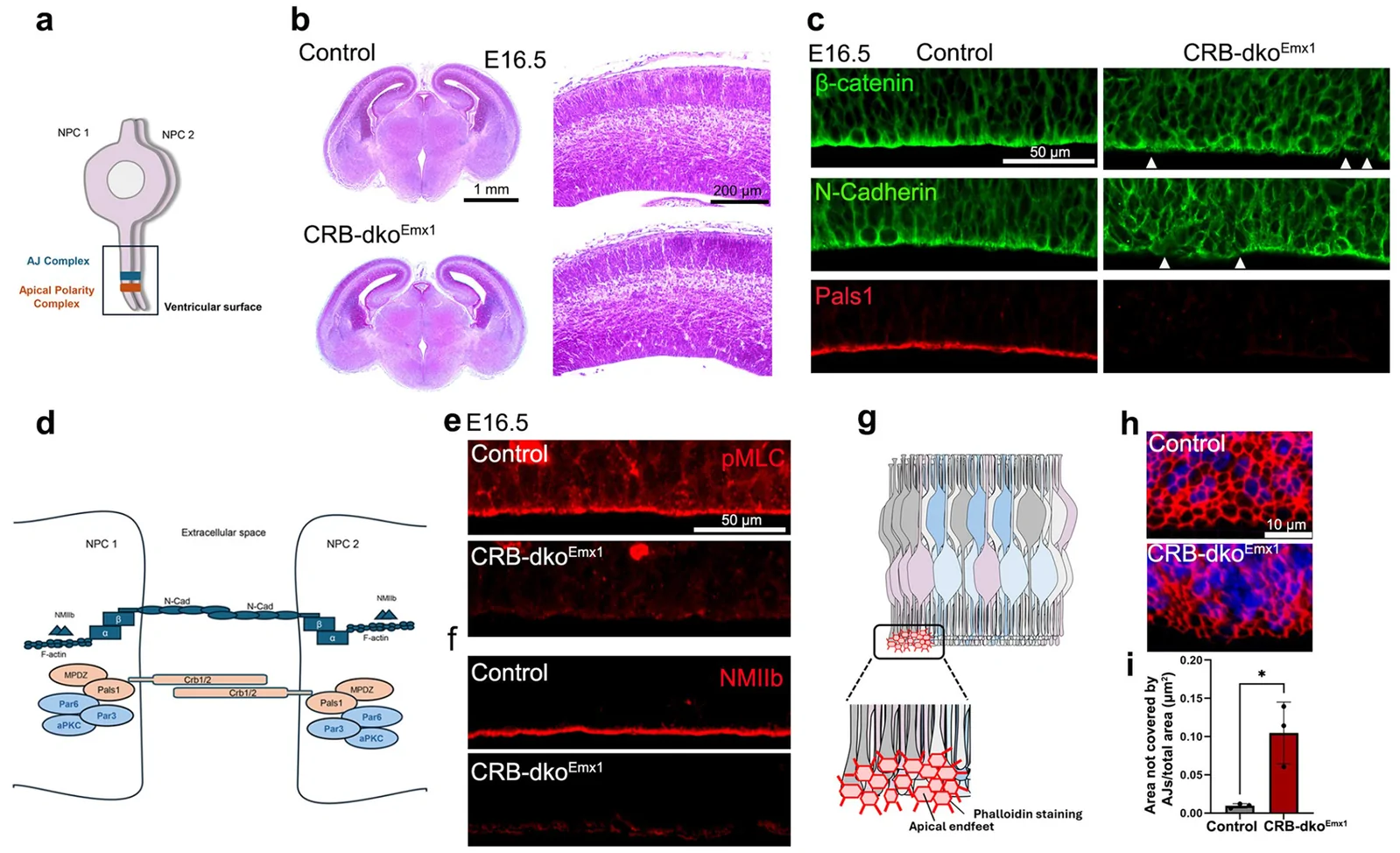
Crb2 loss in cortical progenitors disrupts adherens junctions (AJs) and ventricular integrity. **(a)** Schematic of neural progenitor cells (NPCs) showing the AJ complex and apical polarity complex at their apical endfeet near the ventricular surface. **(b)** Representative coronal H&E-stained sections at E16.5. (N=7 Control, N=5 CRB-dko^Emx1^). **(c)** Confocal images of E16.5 coronal sections along the lateral ventricular wall from control and CRB-dko^Emx1^ brains immunostained for N-cadherin and β-catenin (AJs) and Pals1 (apical polarity). Control exhibits continuous AJs, CRB-dko^Emx1^ show disruptions of AJs (arrowheads) and significant reduction of apical marker Pals1, (N=7 Control, N=5 CRB-dko^Emx1^). **(d)** the neural progenitor cell (NPC) interface showing adherens junctions (AJs, N-cadherin, β-catenin) and the Crb2/Pals1 polarity complex positioned at apical junctions. **(e)** Apical actomyosin components are reduced in CRB-dko^Emx1^ cortex: phospho-myosin light chain (pMLC) and **(f)** non-muscle myosin IIb (NMIIb) immunostaining at the ventricular surface compared with Control, (N=7 Control, N=5 CRB-dko^Emx1^). **(g)** Schematics of the oblique view showing the apical endfeet of the cells in the ventricular lining and experimental design. **(h)** Immunostaining of vibratome dorsal cortical sections showing disrupted phalloidin labeling of the apical F-actin ring at ventricular endfeet in CRB-dko^Emx1^ mice. **(i)** Quantification of the area not covered by AJs/total area (μm^2^), (N=3 Control, N=3 CRB-dko^Emx1^); P = 0.0153. Two-tailed unpaired t-test, in the plot significance is indicated as *P<0.05. Bars show mean ± SD, points, individual animals.

**Figure 6:**
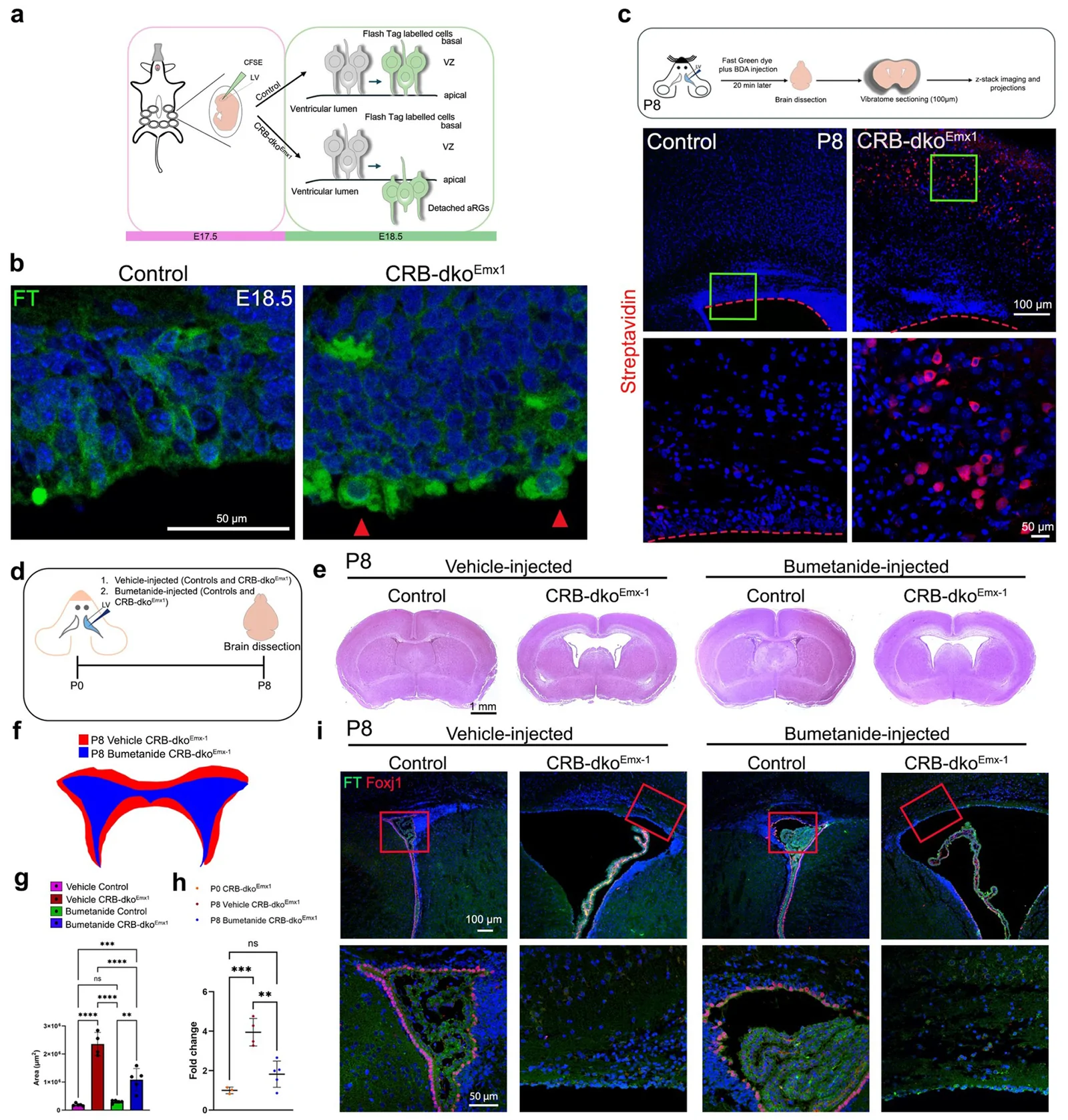
Crb2 loss disrupts ventricular integrity, while bumetanide reduces ventriculomegaly without ependymal rescue. **(a)** Schematic of the FlashTag (FT) procedure. CFSE is injected into the lateral ventricle (LV) at E17.5, and brains are collected at E18.5. **(b)** Representative coronal sections at E18.5 showing FT-labeled ventricular lining cells in Control and in CRB-dko^Emx1^ embryos, red arrowheads indicate displaced FT-positive cells in CRB-dko^Emx1^, (N=3 Control, N=3 CRB-dko^Emx1^). **(c)** Experimental workflow for biotinylated dextran amine (BDA) injection, tissue processing, staining, and imaging and representative dorsal cortical sections of projected z-stacks showing streptavidin signal detected in Control and CRB-dko^Emx1^ embryos (boxed regions shown at higher magnification), (N= 3 Control, N=3 CRB-dko^Emx1^). **(d)** Experimental timeline: at P0, mice received (i) a vehicle injection, or (ii) a bumetanide injection, and brains were collected at P8. **(e)** Representative coronal brain sections at P8 H&E-stained from Control and CRB-dko^Emx1^ mice injected with vehicle or bumetanide. **(f)** Overlay of segmented lateral ventricles. **(g)** Quantification of ventricular area for the indicated groups; (N=5 Vehicle Controls, N=4 Vehicle CRB-dko^Emx1^, N=5 Bumetanide Controls, N=5 Bumetanide CRB-dko^Emx1^). P = 0.0007 (Vehicle Controls vs. Bumetanide CRB-dko^Emx1^); P = 0.0023 (Bumetanide Controls vs. Bumetanide CRB-dko^Emx1^); P = 0.9270 (Vehicle Controls vs. Bumetanide Controls; ns). **(h)** Quantification of ventricular area across indicated groups. Each dot represents one animal. All values are normalized to P0 CRB-dko^Emx1^. P8 CRB-dko^Emx1^ groups received vehicle or bumetanide injection at P0 and were harvested at P8. (N=3 P0 CRB-dko^Emx1^, N=4 P8 Vehicle CRB-dko^Emx1^, N=5 P8 Bumetanide CRB-dko^Emx1^); P = 0.0003 (P0 CRB-dko^Emx1^ vs. P8 Vehicle CRB-dko^Emx1^); P = 0.0014 (P8 Vehicle CRB-dko^Emx1^ vs. P8 Bumetanide CRB-dko^Emx1^); P = 0.2048 (P0 CRB-dko^Emx1^ vs. P8 Bumetanide CRB-dko^Emx1^; ns). **(i)** Representative immunostainings of LV of the brains showing FT (green) with Foxj1 (red) and Hoechst (blue) in vehicle and bumetanide injected mice as indicated. Boxed regions are shown at higher magnification below. (g,h) One-way ANOVA with post-hoc tests. In the plots significance is indicated as **P<0.01, ***P< 0.001, ****P < 0.0001, ns, not significant. Bars show mean ± SD, dots represent individual animals.

**Figure 7:**
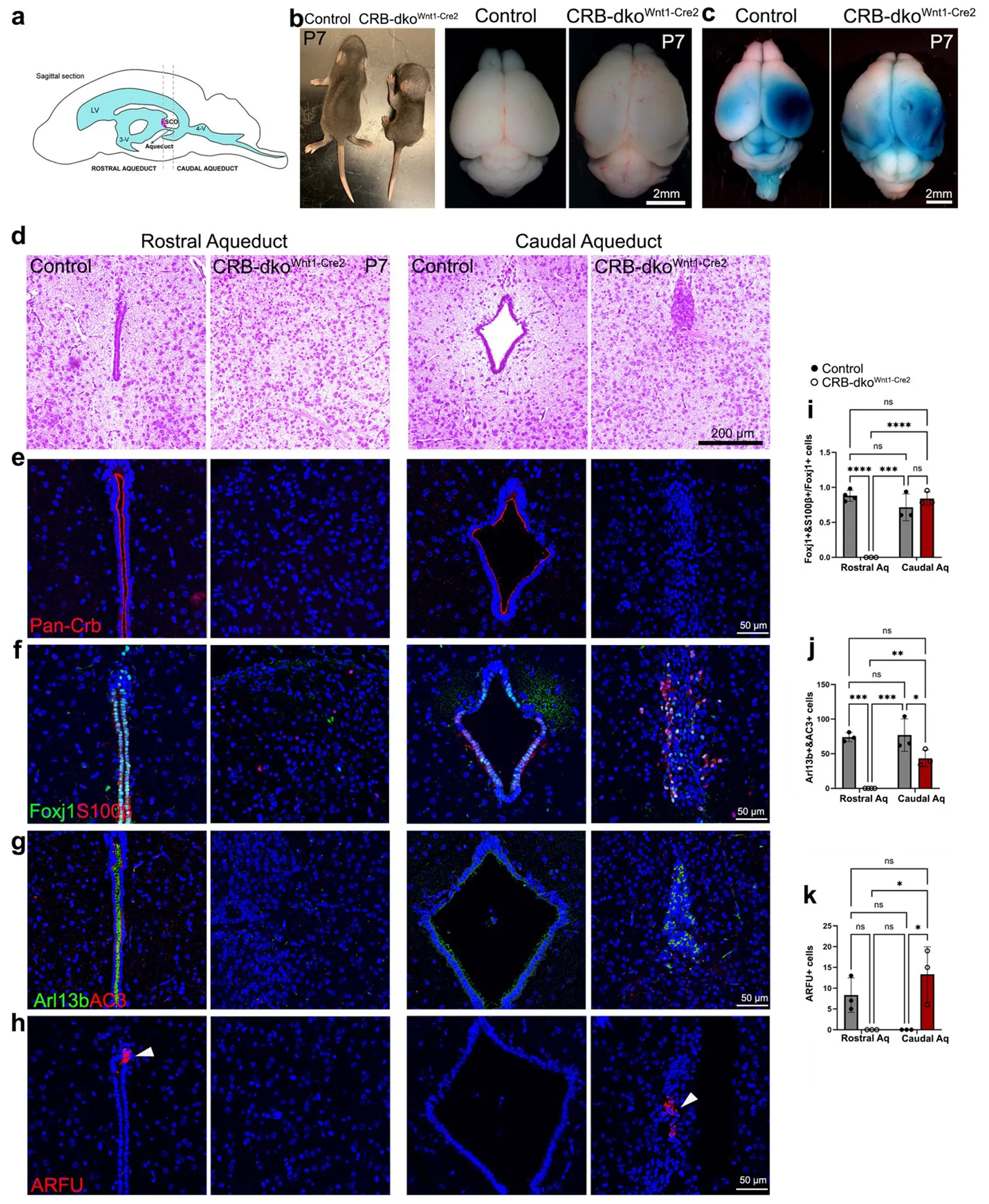
Crb2 loss in midbrain disrupts aqueduct integrity but does not prevent generation of multiciliated ependymal cells. **(a)** Sagittal brain schematic showing the ventricular system, subcommissural organ (SCO), and rostral-to-caudal aqueduct regions at P7. **(b)** Gross morphology and dorsal brain views show domed head and abnormal brain morphology in CRB-dko^Wnt1-Cre2^ mice, consistent with hydrocephalus. **(c)** Fast Green dye intracerebroventricular (ICV) injection reveals impaired CSF flow and marked ventricular enlargement in mutant brains compared with controls. **(d)** Representative coronal H&E-stained sections through the rostral and caudal aqueduct regions (Control vs CRB-dko^Wnt1-Cre2^). Representative coronal sections stained for **(e)** Pan-Crb of the rostral and caudal aqueduct regions, **(f)** Foxj1 (ependymal lineage marker, green) and S100β (mature ependymal marker, red), **(g)** Arl13b and AC3 to assess ciliated ependymal cells, **(h)** AFRU (SCO/Reissner fiber-associated cells marker), Control animals show the expected distribution, whereas mutants show altered localization, with signal reduced rostrally and detected ectopically/accumulated caudally (arrowheads). **(i)** Quantification at P7 of Foxj1^+^&S100β^+^/ Foxj1^+^ cells per section, (rostral aqueduct: N=4 Control, N=3 CRB-dko^Wnt1-Cre2^ mice, caudal aqueduct: N=3 Control, N=3 CRB-dko^Wnt1-Cre2^ mice); P = 0.0001 (Rostral CRB-dko^Wnt1-Cre2^ vs. Caudal Controls); P = 0.2790 (Rostral Controls vs. Caudal Controls; ns); P = 0.9685 (Rostral Controls vs. Caudal CRB-dko^Wnt1-Cre2^; ns); P = 0.5329 (Caudal Controls vs. Caudal CRB-dko^Wnt1-Cre2^; ns). **(j)** Quantification of AC3^+^&Arl13b^+^ cells per section in rostral and caudal aqueduct regions (rostral aqueduct: N=3 Control, N=4 CRB-dko^Wnt1-Cre2^ mice, caudal aqueduct: N=3 Control, N=3 CRB-dko^Wnt1-Cre2^ mice); P = 0.0002 (Rostral Controls vs. Rostral CRB-dko^Wnt1-Cre2^); P = 0.0001 (Rostral CRB-dko^Wnt1-Cre2^ vs. Caudal Controls); P = 0.9912 (Rostral Controls vs. Caudal Controls; ns); P = 0.0665 (Rostral Controls vs. Caudal CRB-dko^Wnt1-Cre2^; ns); P = 0.0073 (Rostral CRB-dko^Wnt1-Cre2^ vs. Caudal CRB-dko^Wnt1-Cre2^) ; P = 0.0432 (Caudal Controls vs. Caudal CRB-dko^Wnt1-Cre2^). **(k)** Quantification of AFRU^+^ cells in rostral and caudal aqueduct regions, (rostral aqueduct: N=3 Control, N=3 CRB-dko^Wnt1-Cre2^ mice, caudal aqueduct: N=3 Control, N=3 CRB-dko^Wnt1-Cre2^ mice); P = 0.1170 (Rostral Controls vs. Rostral CRB-dko^Wnt1-Cre2^; ns); P = 0.1170 (Rostral Controls vs. Caudal Controls; ns); P = 0.4498 (Rostral Controls vs. Caudal CRB-dko^Wnt1-Cre2^; ns); P > 0.9999 (Rostral CRB-dko^Wnt1-Cre2^ vs. Caudal Controls); P = 0.0135 (Rostral CRB-dko^Wnt1-Cre2^ vs. Caudal CRB-dko^Wnt1-Cre2^); P = 0.0135 (Caudal Controls vs. Caudal CRB-dko^Wnt1-Cre2^). **(i,j,k)**: Two-way ANOVA with post-hoc tests. In the plots significance is indicated as *P<0.05, **P<0.01,***P< 0.001, ****P < 0.0001, ns, not significant. Data are presented as mean ± SD.

## Data Availability

All data supporting the findings of this study are available within the article and its additional files. The processed bulk RNA-sequencing count matrix is provided in additional file 10. The raw bulk RNA-sequencing reads are not publicly available at the time of initial submission. The accession number and persistent repository link will be provided during peer review and before publication.
